# Quantum control of exciton wave functions in 2D semiconductors

**DOI:** 10.1126/sciadv.adk6369

**Published:** 2024-03-20

**Authors:** Jenny Hu, Etienne Lorchat, Xueqi Chen, Kenji Watanabe, Takashi Taniguchi, Tony F. Heinz, Puneet A. Murthy, Thibault Chervy

**Affiliations:** ^1^ Department of Applied Physics, Stanford University, Stanford, CA 94305, USA.; ^2^ SLAC National Accelerator Laboratory, Menlo Park, CA 94025, USA.; ^3^ NTT Research, Inc. Physics & Informatics Laboratories, 940 Stewart Dr, Sunnyvale, CA 94085, USA.; ^4^ Research Center for Functional Materials, National Institute for Materials Science, 1-1 Namiki, Tsukuba 305-0044, Japan.; ^5^ International Center for Materials Nanoarchitectonics, National Institute for Materials Science, 1-1 Namiki, Tsukuba 305-0044, Japan.; ^6^ Institute for Quantum Electronics, ETH Zürich, CH-8093 Zürich, Switzerland.

## Abstract

Excitons—bound electron-hole pairs—play a central role in light-matter interaction phenomena and are crucial for wide-ranging applications from light harvesting and generation to quantum information processing. A long-standing challenge in solid-state optics has been to achieve precise and scalable control over excitonic motion. We present a technique using nanostructured gate electrodes to create tailored potential landscapes for excitons in 2D semiconductors, enabling in situ wave function shaping at the nanoscale. Our approach forms electrostatic traps for excitons in various geometries, such as quantum dots, rings, and arrays thereof. We show independent spectral tuning of spatially separated quantum dots, achieving degeneracy despite material disorder. Owing to the strong light-matter coupling of excitons in 2D semiconductors, we observe unambiguous signatures of confined exciton wave functions in optical reflection and photoluminescence measurements. This work unlocks possibilities for engineering exciton dynamics and interactions at the nanometer scale, with implications for optoelectronic devices, topological photonics, and quantum nonlinear optics.

## Teaser

Nanostructures enable control of excitons in 2D materials, offering a scalable approach for quantum optoelectronic applications.

## INTRODUCTION

The ability to trap and manipulate the quantum mechanical state of particles is a central pillar across various disciplines of quantum science, including ultracold atoms, ion traps, and superconducting qubits (*1*–*4*). In semiconductor optics, the traditional approach to confinement of optical excitations involves nanostructures such as self-assembled or colloidal quantum dots, which are typically fabricated through growth or implantation techniques. These methods lead to ensembles of quantum dots (or rings) with random positions and broad energy distribution with limited local control (*5*–*7*). The resulting lack of scalability has hindered their widespread technological implementation. In addition to these methods, alternative material modulation approaches have recently been explored, such as strain engineering (*8*–*12*), electron and ion beam irradiation (*13*, *14*), and moiré potential engineering (*10*, *15*–*17*), which offer varying degrees of control on the positions, energies, or number of emitters. Furthermore, electrostatic confinement of spatially indirect excitons in semiconductor heterostructures has been reported (*18*, *19*); however, their very weak coupling to light limits potential photonic applications. The ability to precisely control the individual quantum states of optically active excitons with strong light-matter coupling has thus remained a major experimental challenge.

Recently, a technique has been reported for confining direct excitons purely using electric fields and charge density gradients in two-dimensional (2D) semiconductor heterostructures (*20*). The method relies on the fact that in-plane electric fields [$\vec{F}\left(r\right)$] induce a quadratic DC Stark shift of the excitons, and charge density [ρ(**r**)] leads to an interaction-induced density-dependent energy shift, according to

$$\Delta E=-\frac{1}{2}\alpha |\vec{F}\left(r\right)|2+\beta \rho \left(r\right)$$ (1)

where α is the DC polarizability of excitons and β is the exciton-electron coupling constant. Spatial variations of doping densities and electric fields can thus be used to trap excitons. On the basis of this effect, quantum confinement was demonstrated in a gate-defined lateral p-i-n junction. However, as the trapping occurs along the gate edges, the technique has so far been limited to excitonic 1D quantum wires.

**Fig. 1.**
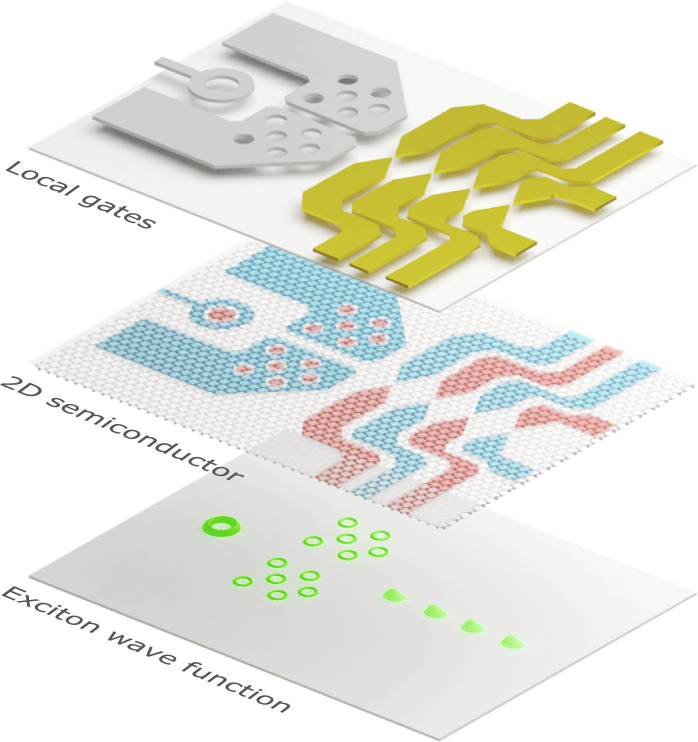
Quantum excitonics in 2D semiconductors. Schematic illustration of our approach. Top layer: Nanostructured gate electrodes are positioned in the vicinity (tens of nanometers) of a 2D semiconductor. This is done by either metal deposition (gold electrodes) or graphite etching (gray electrodes). Middle layer: Voltages applied to the gates define lateral distributions of in-plane electric fields $\vec{F}\left(r\right)$ and charge densities ρ(**r**) for itinerant holes (red) and electrons (blue) in the 2D semiconductor. Bottom layer: Exciton confinement occurs due to a combination of electric field–induced Stark shift and exciton-charge interactions (see Eq. 1). This enables in situ control of the c.o.m exciton wave function in arbitrary traps such as excitonic rings, quantum dots, and scalable arrays.

Here, we demonstrate scalable and tunable electrostatic traps of arbitrary shapes for direct excitons in monolayer transition metal dichalcogenide (TMD) semiconductors. We illustrate our approach in Fig. 1. The crux of our work lies in using lithographically nanostructured gate electrodes in proximity to the 2D semiconductor plane (upper layer). This allows to precisely define the lateral distribution of in-plane electric fields $\vec{F}\left(r\right)$ and density ρ(**r**) of itinerant holes (red) and electrons (blue) in the 2D semiconductor, with a resolution down to a few tens of nanometers (middle layer). This, in turn, enables tailor-made landscapes for excitons with a high degree of control on the wave function profile (lower layer). To highlight the versatility of our technique, we focus on two exemplary trap geometries, namely, excitonic rings and quantum dots, that may serve as building blocks for more extended landscapes. Furthermore, we demonstrate the unprecedented scalability of our approach by realizing arrays of rings and independently controlled quantum dots, which is of particular relevance for future optoelectronic and photonic technologies.

The basic structure of our exciton trapping devices consists of a monolayer TMD semiconductor, such as MoSe_2_, encapsulated by hexagonal boron nitride (hBN) spacers of appropriate thickness. This heterostructure is stacked on a Si/SiO_2_ substrate with top (TG) and bottom (BG) gate electrodes, which can either be graphene (Gr) or metallic thin films. We nanostructure one of the gate electrodes through a combination of electron beam lithography and dry etching, which allows for patterning resolutions of around 50 nm. A global BG is used to define the charge configuration of the entire monolayer, whereas the nanostructured TGs enable local control of charge densities and fields. Although either of the gates can be patterned without losing functionality, we choose to define the structures on the TGs and keep the BG for global doping, for ease of fabrication. Our devices are cooled down to ∼5 K in a closed cycle dry cryostat with optical access. To characterize the excitonic states in our system, we mainly rely on optical broadband reflection and photoluminescence (PL) spectroscopy with a spatial resolution of 0.7 μm. A detailed account of our fabrication and experimental procedures is described in Materials and Methods.

## RESULTS

### Excitonic rings

**Fig. 2.**
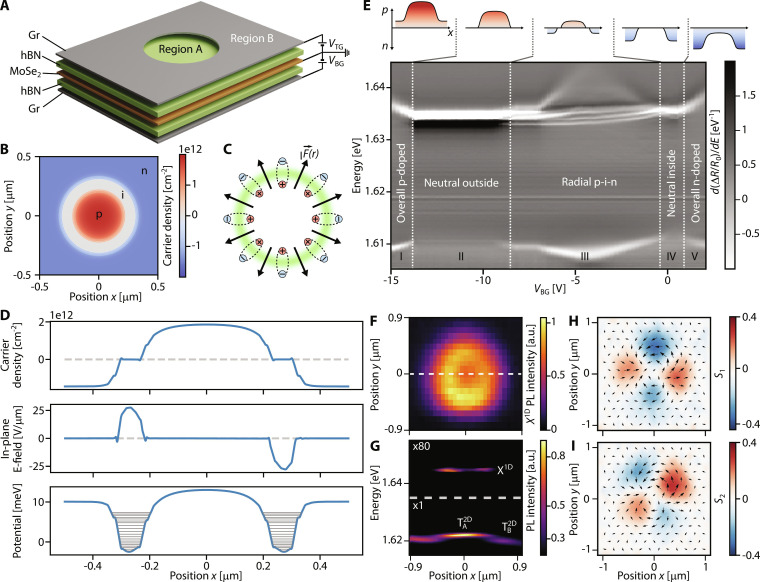
Ring traps for excitons. (**A**) Dual-gated TMD heterostructure where a nanohole etched in the TG defines quantum ring confinement for excitons. (**B**) Finite element electrostatic simulations of the device for a 600-nm gate hole with gate voltage configuration (*V*_BG_, *V*_TG_) = (−5.0 V,9.5 V). Charge distribution shows a globally electron-doped semiconductor with holes trapped in the center of the nanohole, surrounded by a ring-shaped neutral region. (**C**) Schematic showing radially dipolar excitons quantum-confined in a ring. (**D**) Finite element simulations of the charge distribution (top), field distribution (middle), and exciton trapping potential (bottom) for a 600-nm-diameter hole device. The horizontal gray lines in the lower panel are the c.o.m eigenstates of the 2D Schrödinger equation. Gate voltage configuration is the same as in (B). (**E**) First-derivative reflectance contrast spectra as a function of *V*_BG_, for fixed *V*_TG_ = 9.5 V. Charging configurations for each regime (I to V) are illustrated above. (**F**) Spatial scan map of PL emission from the confined states *X*^1D^ on a 1-μm-diameter hole. (**G**) Spectral crosscut through the center of the hole (*y* = 0) along the white dashed line in (F), showing the trion emission from regions A and B, $T_{\text{A}}^{2\text{D}}$ and $T_{\text{B}}^{2\text{D}}$ , and the confined *X*^1D^ emission. PL spectra are obtained under 720-nm illumination at 10 μW. (**H** and **I**) Stokes vector analysis of the confined state emission map in the linear polarization basis, which demonstrates that PL emission of ring states is polarized in the radial-azimuthal basis. Arrows represent the local linear Stokes vector *S* = (*S*_1_, *S*_2_).

In Fig. 2, we describe our scheme to create ring traps for excitons. An annular confinement geometry can be obtained by patterning circular holes in the TG, as shown schematically in Fig. 2A. To obtain the strongest confinement, we apply opposite voltages on the TG and BG to reach the annular p-i-n regime, where a central p-doped puddle is enclosed in a Fermi sea of electrons with a ring-shaped neutral region separating them, as seen in Fig. 2B. The combination of in-plane fields and charge gradients leads to tight confinement of excitons in this annular region (Eq. 1). Furthermore, the in-plane fields lead to radially polarized in-plane dipolar excitons as illustrated in Fig. 2C.

In Fig. 2D, we show results of finite element simulations of the charge and field distributions in the annular p-i-n configuration for a TG hole with diameter 600 nm, which provides a quantitative understanding of the electrostatics of the device. We calculate the confinement potential according to Eq. 1, which shows the expected Mexican hat-like profile. We note that the dominant contribution to the potential comes from the exciton-electron interaction (second term in Eq. 1), whereas the Stark shift has a relatively mild effect. Solving the 2D Schrödinger equation in the center-of-mass (c.o.m) frame of excitons, we obtain discrete radial trap levels, with a level separation of *ℏ*ω ∼ 0.3 to 0.8 meV. We present a detailed account of the trap profile as a function of voltage in Supplementary Text (section F).

To verify the nature of excitonic states in this system, we now move on to optical measurements of the device. In monolayer MoSe_2_, the onset of electron or hole doping is associated with the emergence of a bound trion state and a density-dependent blue shifting exciton state (repulsive polaron) (*21*–*23*). Therefore, measuring reflectance spectra as a function of *V*_TG_ and *V*_BG_ allows to precisely distinguish doping configurations inside and outside the TG hole. Region A (inside the hole) is primarily affected by the BG, whereas region B (outside the hole) can be doped using both TG and BG.

In Fig. 2E, we present the measured reflectance spectra obtained from a 600-nm-diameter hole as a function of *V*_BG_, for a fixed *V*_TG_ = 9.5 V. To highlight the faint signatures of quantum-confined states, the reflectance contrast Δ*R*/*R*_0_ is differentiated with respect to energy as detailed in Supplementary Text (section B). Five distinct regimes can be identified as the applied back gate voltage *V*_BG_ is swept. For positive *V*_BG_, the action of the back gate reinforces that of the TG, leading to overall n-doping of the device (regime V in Fig. 2E). As expected, we observe a trion branch at ∼1.610 eV and a repulsive polaron branch at ∼1.635 eV. Decreasing *V*_BG_ reduces the electron density, until we reach the i-i-n regime where region A is neutral (regime IV), as evidenced by the transfer of oscillator strength from the trion branch to the neutral exciton state. As we decrease *V*_BG_ further, we reach the p-i-n regime where region A is p-doped and region B is n-doped (regime III), with an annular neutral region in between. Here, we observe repulsive polaron branches from both A and B regions, associated to the two types of carriers. In addition, we observe narrow and discrete resonances emerging from the neutral exciton state. We attribute these resonances to 1D ring states of different radial mode orders existing at the periphery of the nanohole. Upon decreasing the BG voltage even further, the confined exciton states merge back with the 2D exciton continuum, as the region B outside the dot reaches charge neutrality (regime II). The observed energy separation between the discrete states is approximately *ℏ*ω ≈ 0.5 meV, in agreement with the numerical simulations (Supplementary Text, section D), corresponding to a harmonic oscillator length of  $𝓁=\sqrt{\hbar /m\omega }∼10$ nm, where *m* is the total exciton mass.

To investigate the spatial profile of the trap, we now turn to a larger TG hole of diameter 1 μm, which is large enough to optically resolve the different regions A and B with a confocal microscope setup. Figure 2F shows a spatially resolved PL map of the hole in the radial p-i-n configuration (*V*_BG_ = −2.5 V, *V*_TG_ = +9.0 V). The emission is filtered at the energy of the confined states. As expected, we observe a clear doughnut-shaped emission profile. The PL spectra taken along a crosscut through the center of the hole (Fig. 2G) show distinct trionic emission from regions A and B, and confined state emission from the annular neutral region.

Further insights into the excitonic ring states can be obtained by studying the polarization texture of the emitted photons. Previous studies on 1D confined states have shown that electron-hole exchange interaction, coupled with tight confinement of the excitonic wave function along one direction, leads to strong polarization splitting in a linear basis (*20*). Applying this argument to our circular geometry, the ring confinement should induce polarization splitting in the radial-azimuthal basis, with the low energy azimuthal state dominating the PL emission. To investigate this behavior, we perform spatially resolved Stokes vector polarimetry on the confined excitonic ring emission. The results, shown in Fig. 2 (H and I), indicate twofold symmetric patterns, both in the horizontal-vertical basis (*S*_1_) and in the diagonal–anti-diagonal basis (*S*_2_), as expected for an azimuthally polarized emitter. The spatial spin texture *S* = (*S*_1_, *S*_2_) reveals a pair of monopoles that we attribute to the existence of a finite *x* − *y* strain in the sample (*24*). These observations demonstrate the quantum confinement of excitons in ring-shaped potentials and constitute the first major result of this work.

### Tunable quantum dots

**Fig. 3.**
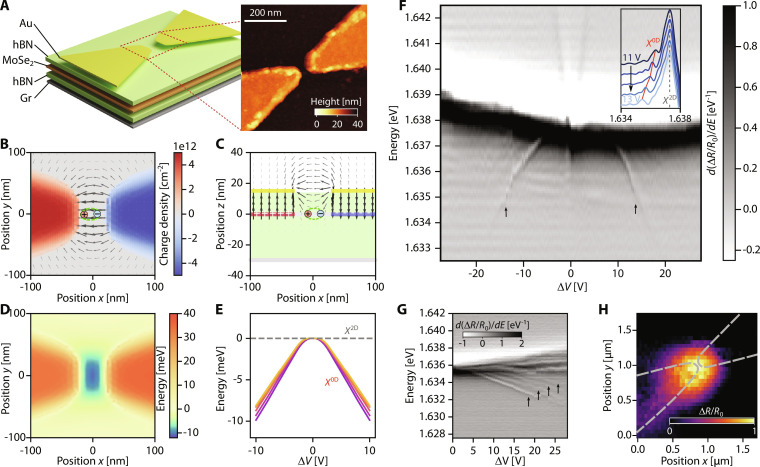
Bow tie traps for excitons. (**A**) Dual-gated TMD heterostructure with TG structured as a bow tie. Inset: AFM map of a 35-nm bow tie gate. (**B** to **D**) Electrostatic simulations with (*V*_L_, *V*_R_, *V*_BG_) ≡ (5.0 V, −5.0 V, 0 V). (B) Top view (*xy*) of charge density and in-plane electric field at the TMD plane, showing n-doped (blue) and p-doped (red) regions. (C) Side view (*xz*) of the electric field at a vertical cut along *y* = 0. (D) Confining potential for excitons in the gap region. (**E**) Calculated dependence of resonance energy of ladder of states as a function of Δ*V* = *V*_L_ − *V*_R_. (**F**) First-derivative reflectance contrast spectroscopy of the bow tie gap region, as a function of Δ*V*. The tunable 0D confined states are pointed out by the arrows. The inset shows vertical crosscuts from Δ*V* = 11 V (dark blue) to Δ*V* = 13 V (light blue). The red line is a guide to the eye following the confined state. (**G**) First-derivative reflectance contrast of a 100-nm gap bow tie, as a function of Δ*V*, showing a ladder of confined states (pointed out by the arrows). (**H**) Spatial dependence of the first-derivative reflectance contrast, integrated over the 0D exciton signal at Δ*V* = 9.9 V. Here, *V*_BG_ = 1.5 V is applied to further energetically separate the 0D state from the *X*^2D^ signal. This leads to a slight elongation of the states toward the left electrode (Supplementary Text, section H).

We now turn to the demonstration of tunable quantum dot-like confinement, which has been a major goal in quantum photonics (*25*–*27*). To generate electrostatic 0D nanotraps, we need a gate geometry that allows for tight confinement in both lateral directions. While the gate hole structure described in Fig. 2 may also be used for this purpose, the tightness of confinement and energy tunability is limited by fabrication constraints. To achieve tunable 0D traps, we design a bow tie electrode structure as illustrated in Fig. 3A, which concentrates electric fields in the nanoscopic gap between the electrodes. These bow ties are fabricated using electron beam lithography and deposition of 10-nm Au, with gap sizes ranging from 30 to 100 nm. An atomic force microscope (AFM) micrograph of a bow tie (∼35-nm gap) is shown in the inset.

In this geometry, we operate primarily in the neutral regime, where the semiconductor is globally set to neutrality using the BG, and a bias voltage Δ*V* = *V*_L_ − *V*_R_ is applied between the bow tie electrodes to obtain in-plane fields in the nanogap. An electrostatic simulation of the charge and field distributions in the device is shown in Fig. 3 (B and C) (top and side view, respectively; red: holes, blue: electrons). In general, the top hBN thickness should be as small as possible (≲20 nm) to maximize the in-plane component of the electric field on the TMD plane in the gap region.

In Fig. 3D, we show the expected 2D trapping potential for excitons in bow ties computed from Eq. 1. First, we note that in contrast to the ring traps, the dominant contribution to confinement in the bow tie geometry comes from the electric field-induced Stark shift (first term in Eq. 1). Further, the trapping potential features *x* − *y* anisotropy, which can be adjusted by varying the width of the electrode tips and the gap size (see Supplementary Text, section K). By solving the 2D Schrödinger equation for the exciton c.o.m motion in such a potential, we obtain the expected dependence of the quantum-confined state energies on Δ*V*, shown in Fig. 3E.

To experimentally observe quantum dot states in the bow tie structure, we measure reflectance spectra in the gap region as a function of Δ*V*. These spectra are acquired from a bow tie with a gap size of ∼35 nm. Since the gap size is much smaller than the diffraction limit, we expect that any confined states will have a strongly diminished oscillator strength as compared to the 2D exciton resonance, dictated by the ratio of the confinement area over the area of the optical spot. In Fig. 3F, we show the first-derivative reflectance contrast spectra *d*(Δ*R*/*R*_0_)/*dE* as a function of Δ*V*. For simplicity, we apply symmetric voltages on the two bow tie electrodes; e.g., to obtain Δ*V* = 20 V, we apply *V*_L_ = −10 V and *V*_R_ = +10 V. In addition to the broad neutral 2D exciton background (centered around *X*^2D^ ∼ 1.638 eV) and the blue-shifting polaron branches from the gated regions, we observe much narrower resonances emerging below the neutral exciton (linewidth Γ ≲ 300 μeV). These states red shift with Δ*V*, with a similar dependence to Fig. 3E. This observed voltage dependence is in excellent quantitative agreement with our simulations (see Supplementary Text, section G). Line cuts at different Δ*V* are shown in the inset. The energy dependence of these states on the electric field across the bow tie is strong evidence that they occur in the gap region (see Supplementary Text, sections G and H). A further compelling signature of quantum confinement in such electrical traps is the emergence of a ladder of states corresponding to discrete energy levels in the trap (*20*). In Fig. 3F, such signatures are barely apparent possibly due to the small gap size leading to vanishing oscillator strength of excited states. To confirm this, we perform the same reflectance measurement on a bow tie with a larger gap size (∼100 nm), as shown in Fig. 3G. Here, we do observe a clear ladder of excitonic states emerging below the 2D exciton continuum, which supports our hypothesis.

To further confirm that the observed resonances originate in the gap region, we perform a spatially resolved scan of reflectivity in the vicinity of the bow tie. For this measurement, a small voltage *V*_BG_ = 1.5 V is applied to further energetically separate the 0D state from the *X*^2D^ background, which facilitates the analysis (see Supplementary Text, section H). The first-derivative reflectance contrast map, integrated over the narrow exciton resonance, is shown in Fig. 3H, which demonstrates that this resonance only appears close to the gap region and vanishes as we move away. We have reproduced these observations across different bow ties in the same device, as well as on several different devices (see Supplementary Text, section J), which underlines the robustness of our technique.

As the confinement area of excitons (*A*) reduces, the repulsive interaction shift (*U*) between excitons is expected to increase as $U\propto E_{B}a_{B}^{2}/A$ (*28*), eventually leading to the regime of single exciton nonlinearity when *U* > Γ, the confined exciton linewidth. To investigate the nonlinear response of the 0D confined states, we perform power-dependent resonance fluorescence (RF) experiments. As detailed in Supplementary Text (section M), a picosecond pulsed laser resonantly excites the 0D state and the generated RF signal is isolated from the pump by polarization filtering. At power levels corresponding to the creation of one to five excitons per pulse, we observe a strong blue-shift of the confined exciton state, accompanied by a saturation of its RF amplitude, consistent with a nonlinear response in the few excitons regime. Together with the spectroscopic measurements in Fig. 3, our experiments unambiguously demonstrate electrically tunable 0D quantum confinement in bow tie electrode structures, which constitutes the second key result of this work.

### Scalable arrays of quantum dots and rings

**Fig. 4.**
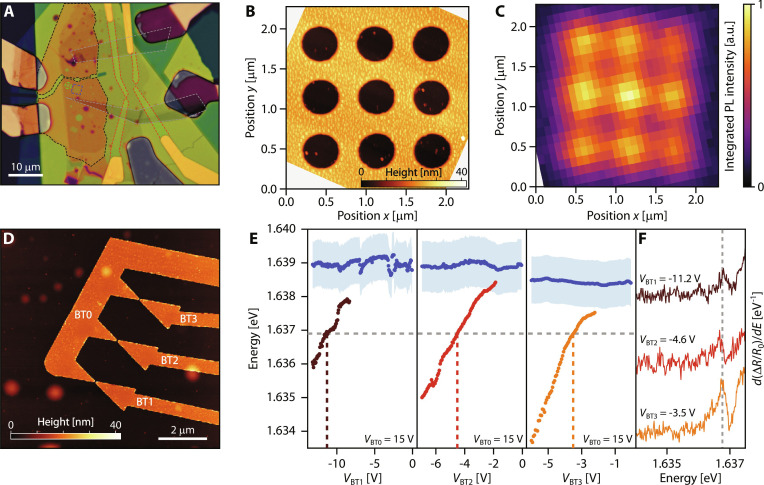
Scalable arrays. (**A**) Optical micrograph of a vdW heterostructure featuring Au bow tie electrodes (red dashed lines) and patterned few layer graphene TG (black dashed lines). The blue dashed line indicates the 400-nm-diameter hole array, and the green one highlights the 1-μm-diameter hole studied in Fig. 2. The gray dashed lines indicate the contacts to the TMD. (**B**) AFM topography of the 400-nm hole array. (**C**) PL map of the hole array, filtered at the confined state emission energy. (**D**) AFM topography of a bow tie array. BT1, BT2, and BT3 are three independent electrodes, while the counter electrode BT0 is common to the three bow ties. (**E**) Fitted energies of the 2D exciton (blue dots) and 0D confined states (brown, red, orange dots) for a fixed counter electrode voltage *V*_BT0_ = 15 V, as a function of the independent electrode voltages (*V*_BT1_, *V*_BT2_, *V*_BT3_). The shaded blue area indicates the FWHM of *X*^2D^. (**F**) Confined state spectra acquired under resonance tuning conditions (*V*_BT0_ = 15 V, *V*_BT1_ = −11.2 V, *V*_BT2_ = −4.6 V, *V*_BT3_ = −3.5 V).

The most important advantage of our approach for electrical nanoscale control of exciton wave functions is the potential for scaling up to more complex structures. The quantum dot and ring trapping schemes that we presented above can be seen as the building blocks for larger systems. Now, we demonstrate how a variety of nanostructures can be realized in a single TMD heterostructure by lithographically defining the desired gate patterns (Fig. 4). In Fig. 4B, we show an AFM scan of a 3 × 3 array of 400-nm-diameter holes, with a 600-nm pitch that allows to optically resolve individual lattice sites. In Fig. 4C, we perform a spatially resolved scan of PL emission integrated over the ring state resonance, similar to Fig. 2F. We observe the emission profile of the ring array that matches excellently with the gate pattern. Since the hole diameters are smaller than the diffraction limit, we do not resolve the ring shape of the emission patterns. This shows that ring traps can be scaled up arbitrarily in a single monolayer.

Next, we demonstrate the scalability of electrically tunable quantum dots. An important motivation for our work is the realization of multiple quantum dots with identical energies, which is a crucial and basic ingredient for several applications, including photonic quantum information processing and quantum communications (*29*). This has so far remained a hurdle to achieve since existing material modulation approaches are drastically limited by material disorder and process variation (*30*). We address this problem by fabricating an array of bow ties with independent control for each. An AFM scan of our structure with three bow ties with approximate gap size of 50 nm and a separation of 1 μm is shown in Fig. 4D. We electrically short the left-hand side electrodes of the three bow ties (BT0) while maintaining individual control on each of the right-hand side electrodes (BT1, BT2, BT3). This allows to reduce the number of control gates per quantum dot to only one, thus enhancing scalability without compromising on the control over each site. As shown in Supplementary Text (section J), we observe the quantum dot confinement signatures described in Fig. 3F in each of the bow ties.

In Fig. 4E, we report the fitted energy of the 0D states as a function of individual gate voltages *V*_BT1_, *V*_BT2_, and *V*_BT3_, while keeping the common counter electrode at *V*_BT0_ = 15 V. The energy of the 2D exciton *X*^2D^ is shown with blue dots, and the linewidth is indicated by the blue shaded regions. The three bow ties show different dependence with voltage, possibly due to material disorder and variations due to fabrication uncertainties. Nevertheless, the three quantum dots can be simultaneously tuned to degeneracy (horizontal dashed line) by applying the suitable voltages across the bow ties (vertical dashed lines). This is evident in the spectra shown in Fig. 4F, taken at *V*_BT1_ = −11.2 V, *V*_BT2_ = −4.6 V, and *V*_BT3_ = −3.5 V, which show three quantum dot states resonantly tuned in energy. The ability to combine position-controlled lithographically defined quantum dots, with simultaneous and independent energy tunability into scalable arrays of quantum sites, constitutes the third main result of this work.

## DISCUSSION

We have demonstrated fundamental building blocks for quantum excitonics. Our approach enables continuous control of the c.o.m wave functions, from micrometer-sized extended states all the way down to quantum confinement into nanoscale dots and rings, and is intrinsically scalable to independently tunable arrays. A key advantage of this method is that arbitrary landscapes for excitons can be defined in a nonintrusive manner while retaining the pristine properties of the active material. Hence, it can be extended to any 2D semiconductor. Furthermore, improvements in fabrication techniques—in particular in lithography—will enable even smaller trapping length scales and better spatial control.

Our work opens up several avenues for further research. On the fundamental level, an important question is the nature of the confined excitonic states in the relative coordinates, which may be investigated by applying a combination of strong electric and magnetic fields (*31*–*35*). Moreover, a detailed study of lifetime and coherence time is necessary to obtain insights into exciton dynamics in such potentials. The fact that excitons confined in such nanoscopic traps still retain substantial oscillator strength is a major advantage, as this will enable coupling such structures to optical microcavities (*36*) and obtain Rabi splitting in the meV range. Arrays of electrically confined quantum dots and rings may be combined with microcavity arrays to realize Bose-Hubbard model of photons. The enhanced nonlinearity of confined exciton systems may allow exploration of strongly correlated many-body states of light. Along the same lines, trapping radially dipolar excitons in ring potentials may have immediate relevance for effecting artificial gauge fields for optical excitations, which is key to realizing topological effects such as a photonic fractional quantum Hall state. From the technological perspective, these configurable exciton landscapes could be of relevance for development of active photonic metamaterials (*37*) and light sources (*38*).

## MATERIALS AND METHODS

### Experimental setup

Our experimental setup is depicted in fig. S1. The sample is kept at ∼5 K using an attoDRY800 closed-loop cryostat from Attocube. The sample is fixed on a custom-made PCB board, which is mounted on a positioning piezo stack composed of three piezo steppers (two Attocube ANPx101/RES/LT and one ANPz102/RES/LT) and a piezo scanner (ANSxyz100std/LT). A cryo-, vacuum-compatible, high–numerical aperture (NA = 0.81) objective (Attocube LT-APO/NIR/0.81) is used within the cryostat space. Voltages are applied to the sample gates using a source measure unit (SMU) from Hewlett-Packard (HP4142B) fitted with HP 41422B (41420A) modules.

The white light reflectivity measurements are performed using a superluminescent light-emitting diode (SLED Exalos: EXS210065-01). The reflectivity contrast (RC) maps, measured using a white light SLED (20-nm spectral bandwidth), show no appreciable changes up to 5 μW of optical power, where photodoping or heating of the sample starts to influence the RC gate maps. All reported RC data were measured at powers below 1 μW. A 720-nm Ti:Sa continuous wave tunable laser (Matisse C from Spectra Physics) is used for PL measurement. The signal beam is collected through a single-mode optical fiber (Thorlabs 630HP) that we use as a confocality pin hole. The corresponding collection area on the sample surface has a full width at half maximum (FWHM) of 0.7 μm, which sets the spatial resolution of our optical setup. The fiber output is directed to an imaging spectrometer (Andor SR-750-D1 with SR5-GRT-0600-0500, 600 line per mm grating) equipped with a Peltier cooled charge-coupled device (CCD) camera (Andor DU940P-UV).

Polarization-resolved experiments are performed using a set of broadband polarization optics in the excitation and detection arms, both consisting of linear polarizers (Thorlabs LPVIS100-MP2), half-wave plates (Thorlabs AHWP10M-980), and quarter-wave plates (Thorlabs AQWP10M-980).

AFM topographies are obtained in tapping mode, using ScanAsyst-air AFM probes on a MultiMode-8-HR AFM from Bruker.

### Sample fabrication

All hBN, graphite, and MoSe_2_ flakes used in this work are produced with mechanical exfoliation using scotch tape. The high-quality hBN crystal is from the National Institute for Materials Science, and the MoSe_2_ crystal is from HQ graphene. The van der Waals (vdW) heterostructure is assembled using the standard PC (polycarbonate) dry transfer method in an ambient environment (*39*). The heterostructure is then dropped down to a SiO_2_/Si substrate at 180°C. Multilayer graphite flakes are picked up as contacts to the MoSe_2_ monolayer.

Nanometer-scale features are generated with e-beam lithography (Raith Voyager, 50 keV) using a thin poly(methyl methacrylate) (PMMA) layer of 100 nm thick. For the excitonic ring samples, the vdW heterostructure consists of hBN capping layer/top Gr gate/top hBN/MoSe_2_ monolayer/bottom hBN/bottom Gr gate. After e-beam patterning, the hBN capping layer and the graphite TG are etched by reactive ion etching (Oxford Plasma Pro 80) sequentially. Ten standard cubic centimeters per minute (sccm) CHF_3_/10 sccm Ar/2 sccm O_2_ is used to etch hBN, and 10 sccm O_2_ is used to etch graphite. For the bow tie samples, the heterostructure consists of top hBN/MoSe_2_ monolayer/bottom hBN/bottom Gr gate. The same e-beam lithography process is used to pattern the nanogap, and then a thin layer of metal (3 nm Ti/10 nm Au) is deposited as the TGs with a Kurt J. Lesker high vacuum e-beam evaporator. The thickness of the hBN layer separating the TMD from the patterned gate layer dictates the minimal scale of confinement achievable on the device, due to the shadowing effect.

The leads and bonding pads to the patterned gates and the sample contacts are formed by 5 nm Ti/45 nm Au, with patterns generated by either e-beam lithography (with 200-nm PMMA) or photo-lithography (ML3 MicroWriter).

To achieve a high yield in observing confined excitons, we first map the reflection contrast of the MoSe_2_ monolayer stack at 4 K to identify homogeneous areas with sharp and uniform A exciton resonance, where we later pattern the nanoholes or bow ties. Following this process, we observed the confined exciton signatures in over 90% of the fabricated devices. Remaining variability in the patterning, etching, and deposition leads to variations in the actual size of the fabricated structures. For instance, we measure gap sizes ranging from 30 nm to 50 nm for nominally 50-nm bow ties, and from 80 nm to 100 nm for nominally 100-nm bow ties. For structures larger than 200 nm, the variation from the e-beam lithography process is usually negligible. Meanwhile, the voltage range where we see the confined states, and the energy of these states can also differ from structure to structure and sample to sample, due to slightly different intrinsic doping, dielectric environment, or strain.

*Note added in proof*: After the manuscript was accepted for publication, the authors alerted the Editorial Office to an additional paper that reported signatures of 0D exciton confinement in similar device structures:

D. Thureja, E. Yazici, T. Smolenski, M. Kroner, D. J. Norris, A. Imamoglu, Electrically defined quantum dots for bosonic excitons. arXiv:2402.19278.
