# Supplementary material for: Quantum control of exciton wave functions in 2D semiconductors
Source: Sci Adv. 2024 Mar 20;10(12):eadk6369. doi: 10.1126/sciadv.adk6369 (PMC10954220; doi:10.1126/sciadv.adk6369)
Supplement: Supplementary file 1 — Supplementary Text Figs. S1 to S15 Table S1 [file sciadv.adk6369_sm.pdf]

Science Advances
AAAS

Supplementary Materials for

Quantum control of exciton wave functions in 2D semiconductors

Jenny Hu *et al.*

Corresponding author: Puneet A. Murthy, murthyp@ethz.ch; Thibault Chervy, thibault.chervy@ntt-research.com

Sci. Adv. **10**, eadk6369 (2024)
DOI: 10.1126/sciadv.adk6369

This PDF file includes:

Supplementary Text
Figs. S1 to S15
Table S1

A Stokes measurement

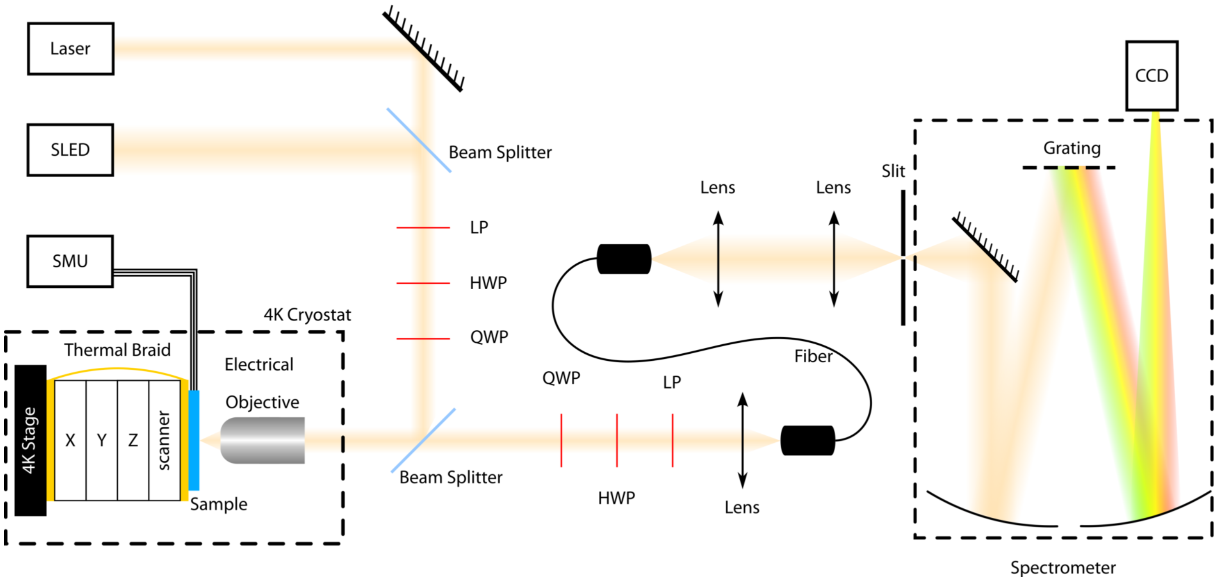

Figure S1: **Schematic of the optical setup.**

Linear Stokes vector maps are obtained by recording PL spectra in different linear polarization basis at each point on the sample:

$$S_0 = \langle E_x^2 \rangle + \langle E_y^2 \rangle = \langle E_d^2 \rangle + \langle E_a^2 \rangle$$
$$S_1 = (\langle E_x^2 \rangle - \langle E_y^2 \rangle)/S_0$$
$$S_2 = (\langle E_d^2 \rangle - \langle E_a^2 \rangle)/S_0$$

where $\langle E_{x(y)}^2 \rangle$ is the intensity of light polarized along x (y) direction, and $\langle E_{d(a)}^2 \rangle$ is the intensity of light polarized along the diagonal (antidiagonal) direction. S_0 is the total intensity of the signal.

B Differential reflectivity data analysis

Differential reflectivity spectra $\Delta R/R_0 = (R - R_0)/R_0$ are obtained from the measured reflectivity R by subtracting and dividing by the background spectrum R_0, measured at the same spot with strong homogeneous doping. This is achieved by keeping the local top gates at 0 V and applying 10 V to the global back gate. Where needed, the differential reflectivity spectra are numerically differentiate with respect to energy ($d(\Delta R/R_0)/dE$) in order to increase the visibility of the confined states resonances.

In Fig. 4 E, the energy of the 2D exciton and 0D exciton are shown. Those are extracted from fits of $d(\Delta R/R_0)/dE$ series. The reflectivity spectral lineshape ($S(E)$) of heterostructures as studied here are well approximated by the sum of a pure and dispersive Lorentzian lineshapes (L_0 and L_D respectively), defined as follow:

$$L_0(E) = \frac{A\Gamma}{2\left[(E-E_0)^2 + (\Gamma/2)^2\right]}$$
$$L_D(E) = \frac{A(E-E_0)}{2\left[(E-E_0)^2 + (\Gamma/2)^2\right]}$$
$$S(E) = \cos(\theta)L_0(E) + \sin(\theta)L_D(E)$$

with A the Lorentzian amplitude, Γ the Full Width at Half Maximum (FWHM), E_0 the central energy of the mode and θ the phase between the pure and dispersive Lorentzian. This Lorentzian is then numerically differentiate and fitted to the data, using the least_squares routine from the python library scipy.optimize.

The spectra present different features: the trapped exciton signal and a broad resonance for the 2D exciton. Three complex Lorentzian are used to fit the spectra, using the same phase parameter θ. Two Lorentzians are needed to fit the 2D exciton, most likely due to local strain in the TMD layer. Fig. S2 present an example of fit for the three different bow ties.

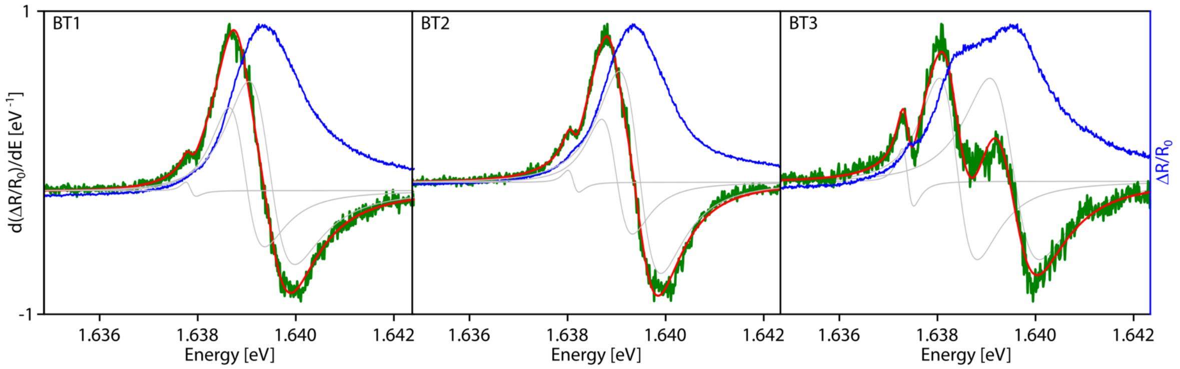

Figure S2: **Representative fitting results for the three different bow ties.** The blue curve is the differential reflectivity spectrum $\Delta R/R_0$, the green one is its energy derivative, $d(\Delta R/R_0)$/dE, and the red one is the triple dispersive Lorentzian fit. The thin grey lines represent each individual derivated Lorentzian used to fit the derivative differential reflectivity.

C Device parameters

The thicknesses of the top and bottom hBN of the devices discussed in the main text is listed in Table S1.

Table S1: **Top and bottom hBN thicknesses of the devices discussed in the main text.**

Relevant figure	top hBN [nm]	bottom hBN [nm]	structures
(Fig.2 B, D, E)	40	40	rings
(Fig. 2 F-I), (Fig. 4 A-C)	40	20	rings, 100 nm bow ties
(Fig. 3), (Fig. 4 D, E)	15	25	50 nm and 100 nm bow ties

D Electrostatic simulation method

The electrostatic simulation is done with COMSOL multiphysics electrostatic modeling on a 3D grid using finite element method. The TMD monolayer is modeled as a semiconductor, sandwiched in two insulating hBN slabs. The bottom gate is global and the top gate is set to be certain geometry. See (*20*) for detailed parameters.

After the trapping potential is obtained from COMSOL simulation, the confined state energy and wavefunction is obtained by solving the 2D Schrödinger equation numerically in the c.o.m frame.

E Doping diagram

Fig. S3 presents a sketch of the top and bottom gate voltage dependence of the doping for a nanohole structure. First, the sample can be separated into two different region, the dual gated region outside the hole (region B in the main text), and the single gated region inside the hole (region A in the main text).

In region A, only the bottom gate acts to set the charge density of the TMD. Therefore, within the hole, the doping will switch from p- to n-doped due to the sole bottom gate. This result in the vertical lines on the doping diagram Fig. S3, which are the bottom gate voltage at which the TMD switch from neutral to p- or n-doped.

The dual gated region however, one can tune separately the vertical electric field and the TMD doping. An opposite voltage between the top and bottom gate will result in an out of plane electric field and no doping, which is the central diagonal line of Fig. S3. On each side of this diagonal, p- and n-doped region of the outter hole appears.

Since the trapping of 1D edge states occurs in the p-i-n regions, the excitonic ring trap only appear in the upper left and bottom right red triangles of the Fig. S3, where the doping in the hole is of a different kind than the doping outside it.

In addition, a weaker trap can be present in theory for the repulsive polaron, when both top gate and bottom gate are positive or negative. In this case, the inner part of the hole would be less doped, which will results in a lower energy polaron in the hole, and a higher one outside. The dashed line in Fig. S3 shows the limit at which the top gate voltage changes it sign which will induce a lower charge density outside of the hole and a larger one within. This latter configuration lead to an anti-trap for the repulsive polaron.

F Trapping potential vs V_{BG} in the 600 nm hole

Fig. S4 shows the simulated trapping potential at different V_{BG} in a 600 nm hole. Here V_{TG} is set to be $9.5\,\mathrm{V}$, the same as in Fig. 2 E. A ring-shaped trap is only formed when V_{BG} is from $-9\,\mathrm{V}$ to $-1\,\mathrm{V}$, which agrees with our observation in Fig. 2 E that the trapped states only show up in regime III.

G Simulation results for tunable 0D states in the bow tie

Electrostatic simulation for a nano-gap structure made with thin metal layers can be tricky. Due to the small size of the electrode and reduced conductivity, the voltage applied from the source-measurement unit might not reflect the actual voltage drop across the bow tie. This results in a much larger energy dispersion in the simulation compared to the measurements.

Here we present a way to calibrate the actual ΔV with respect to the applied ΔV. In the reflectivity spectra in Fig. 3 F, we observe both the 0D states and the blue-shifting repulsive polaron (RP) emerging from charging the TMD below the left and right electrodes. The blueshift of the RP corresponds to the second term in Eq. 1, which can be calculated from the simulated charge density. By comparing it to the measured RP blueshift, we determine a reduction factor that overlaps the simulated polaron shift to the measured polaron shift, as shown in Fig. S5 (here $\Delta V_{\text{actual}} = 0.33 \Delta V_{\text{applied}}$). Then we use the same reduction factor to calculate the trapping potential and solve the energy dispersion of the 0D states (shown in red dots in Fig. S5). It agrees reasonably well with the measurement.

H Transition from 0D confinement to 1D confinement in the bow tie

Here in Fig. S7, we show the simulation results for the potential landscape in the bow tie structure, as a function of the backgate voltage. The left and right bow tie electrodes are at +5 V and -5 V respectively, i.e. $\Delta V = 10\,\mathrm{V}$. It demonstrates a continuous transition from the 0D dot confinement ($V_{\mathrm{BG}} = 0\,\mathrm{V}$) to 1D edge confinement wrapped around the right (left) electrode when V_{BG} is tuned to negative (positive) values. This 0D to 1D transition is observed experimentally as well, see Fig. S8.

In Fig. S9 we show a $V_{\mathrm{L}} - V_{\mathrm{R}}$ dual gate reflection contrast scan at $V_{\mathrm{BG}} = 0\,\mathrm{V}$. Panel A is the integrated reflection map, and the vertical (horizontal) stripe shows the gates range where the 2D exciton under the right (left) bow tie electrode is kept neutral. Panel B is the $\Delta R/R_0$ line cut along the solid line in panel A which corresponds to applying ΔV across the bow tie. As expected, signature for 0D trapped states is present. Panel C is the $\Delta R/R_0$ line cut along the dashed line in panel A, corresponding to $\Delta V = 0\,\mathrm{V}$. No 0D state is observed because no trap is formed when the same voltage is applied to both bow tie electrodes.

In Fig. S10 we show a $V_{\mathrm{L}} - V_{\mathrm{R}}$ dual gate scan map of reflectivity at $V_{\mathrm{BG}} = 10\,\mathrm{V}$. Panel A is the integrated reflection map, and the vertical (horizontal) stripe indicates the gates range where the 2D exciton under the right (left) bow tie electrode is tuned to neutrality. This is at a negative voltage now because V_{BG} is set to be positive. Panel B is the $\Delta R/R_0$ line cut along the solid line at $V_{\mathrm{R}} = 0\,\mathrm{V}$, showing 1D confined states around the left bow tie electrode (as shown in the inset). Panel C is the $\Delta R/R_0$ line cut along the dashed line at $V_{\mathrm{L}} = 0\,\mathrm{V}$, showing 1D confined states around the right bow tie electrode. Panel D plots the line cut along the dashdot line, displaying two groups of 1D confined states around both bow tie electrodes. The two sets of 1D confined states can also be tuned to resonance. This shows that bow tie structures are very versatile and can host both 0D and 1D tunable confined exciton.

We compare the reflection contrast spectra of 2D exciton, 1D confined exciton and 0D confined exciton formed in the same bow tie structure in Fig. S6. The spectra are taken from Fig. S9 and Fig. S10 respectively.

I Polarization dependence of the 0D states

In Fig. S11, we show the polarization dependence of the 0D trapped states in the bow tie structure. The trapped state follows a linear polarization basis, consistent with the anisotropy of the trapping potential shown in Fig. 3 D.

J 0D trapped states in multiple samples

The 0D trapped state is consistently observed in various bow tie structures on multiple samples, as shown in Fig. S13. For some bow ties, we only see 0D state on one side of ΔV, possibly due to charge inhomogeneity around the nano-gap region.

K Shaping the 0D confined state with different bow tie geometry

Here we show that by designing bow ties with different geometries, the confined excitons can be shaped into more symmetric or elongated wavefunctions. In Fig. S13, we simulate and compare the trapping potential and the ground state wavefunction in three different trap geometries with tip width of 20, 50, and 100 nm and a gap size of 50 nm. As shown, a 20 by 50 nm bow tie is expected to give a symmetric ground state wavefunction, while a 100 by 50 nm bow tie will further elongate the exciton wavefunction.

L Photoluminescence from 0D confined states

Fig. S14 shows the gate dependant (ΔV) PL scan of a $100\,\mathrm{nm}$ by $100\,\mathrm{nm}$ bow tie at different excitation powers. At low power ($5\,\mu$W), the dispersion of the 0D states with voltage is in good agreement with the RC spectrum (left panel). At higher optical pump power ($100\,\mu$W), the 0D states exhibits a blueshift, while the 2D exciton remains unaffected. We further investigate this non-linear effect in resonance fluorescence experiments.

M Resonance fluorescence and non-linearity measurements

In order to investigate the effect of 0D confinement on the non-linear response of excitons, we performed ps pulsed resonance fluorescence (RF) experiments. In these experiments, a linearly polarized $1\,$ps pulse ($1.8\,$meV bandwidth, $80\,$MHz repetition frequency) is resonantly tuned to the 0D exciton energy. The RF signal is recorded under cross-polarized detection conditions, with a polarization extinction ratio of $1 : 10^5$. RF spectra from the 0D state, normalized to the respective pump powers, are reported in Fig. S15 A. The corresponding power-dependent central peak energy, as well as the integrated RF intensities are reported in Fig. S15 B as a function of pump power, clearly demonstrating blue-shift and saturation behaviors, as expected from tightly confined 0D exciton states.

The onset of non-linearity at $10-50\,$nW corresponds to a population of 1-5 excitons/pulse. This can be estimated from the pump fluence and the 0D exciton cross-section area. For $1\,$ps pulse duration at $750\,$nm wavelength, and $80\,$MHz repetition rate, $10\,$nW CW equivalent power corresponds to approximately $N \approx 400$ photons/pulse over the entire optical spot. This number should be reduced by the confined exciton cross-section area, given by the ratio of the confinement area over the area of the optical spot $\sim (20\,\mathrm{nm})^2/(0.25\,\mu\mathrm{m})^2 \sim 6 \cdot 10^{-3}$. Therefore, we expect a population of 1-5 excitons populating the 0D trap at these low excitation powers.

In order to evaluate the stability of the 0D state with respect to its fluctuating environment, we recorded high resolution RF spectra over a time period of $10\,$hours. For this experiment, we use a low intensity CW laser ($1.637\,$eV, $5\,$nW) to generate the RF signal, and scan the 0D state energy by ramping the gate voltage ΔV up and down. This measurement is repeated 100 times, over the period of $10\,$hours, allowing to assess the impact of electrostatic fluctuations as well as potential gate hysteresis on the 0D state. The results, shown in Fig. S15 C demonstrate a good stability of the 0D states, with RMS fluctuations of $0.1\Gamma_{\Delta V}$, with $\Gamma_{\Delta V}$ the linewidth of the 0D state along the ΔV axis.

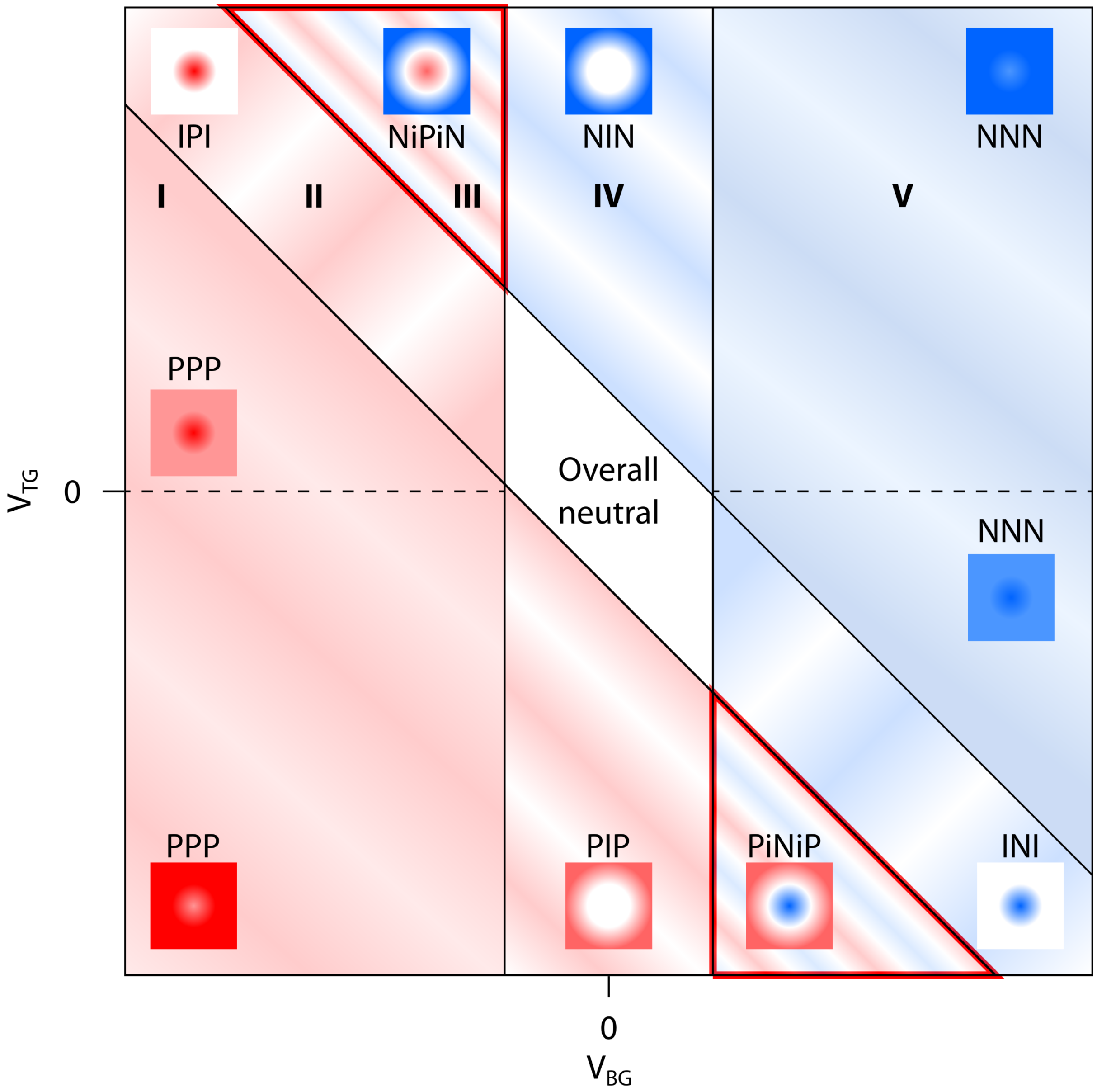

Figure S3: **Doping diagram for a dual gated TMD with a hole in the top gate.** Each region separated by a solid black line correspond to a different doping state of the TMD. This doping state is sketched in the squares, that are charge density map, from hole doped in red to electron doped in blue, passing by white at neutrality. The thick red triangles highlight the PiNiP and NiPiN region, where the TMD 2D exciton can be trapped into a ring. The bold Roman numerals correspond to the doping regime in Fig. 2 of the main text. The two red triangles correspond to the region where ring confinement occurs.

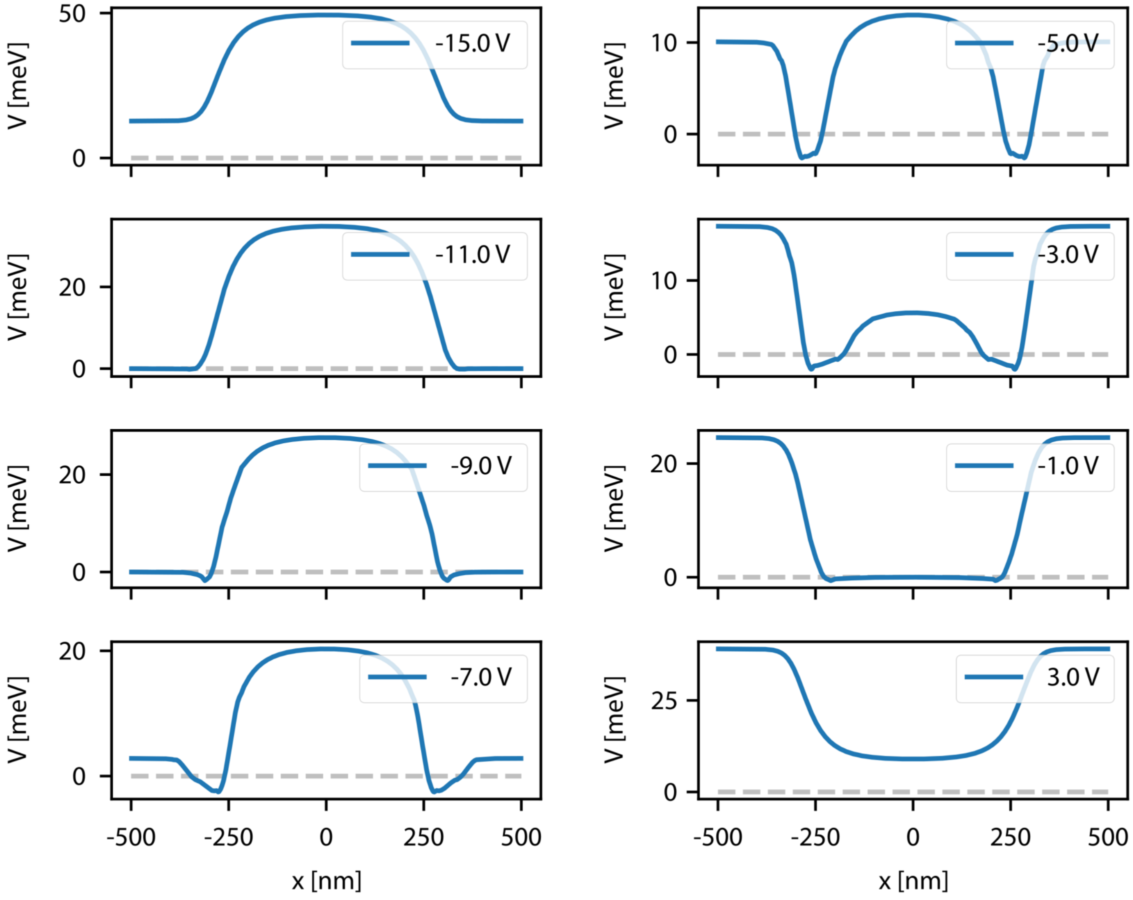

Figure S4: **Electrostatic simulation of the trapping potential at different V_{BG} in the 600 nm hole.** V_{TG} is set to be 9.5 V, the same as in Fig. 2 E.

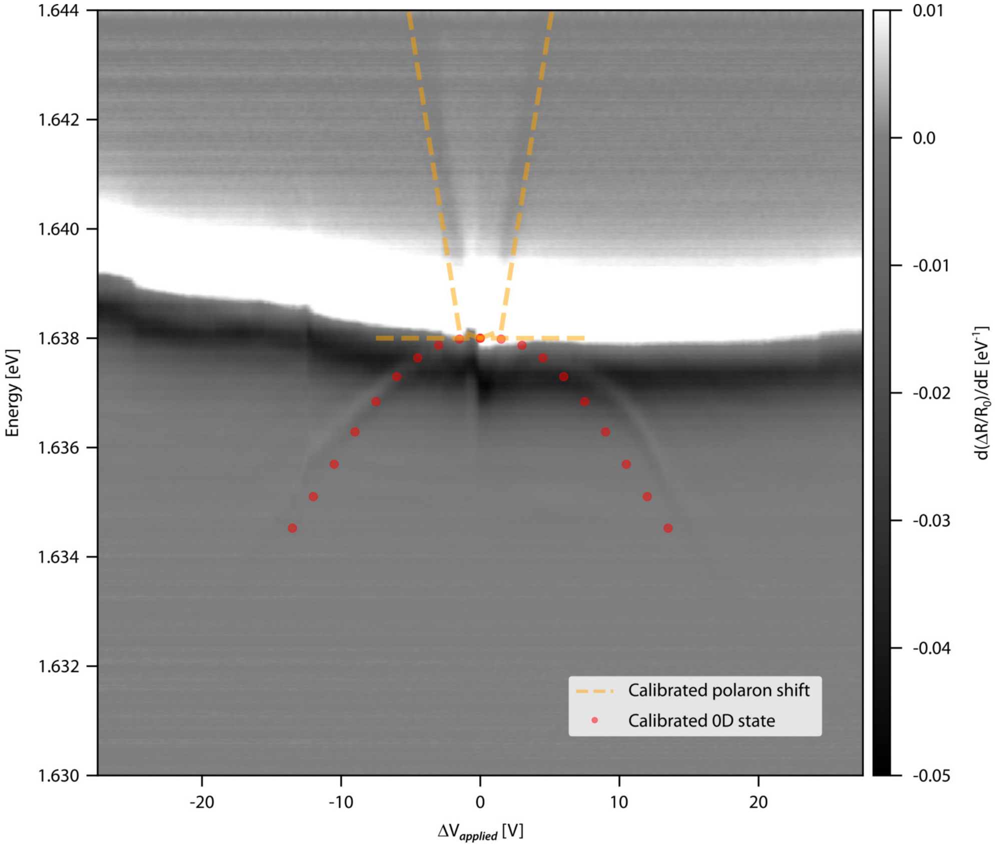

Figure S5: **Comparison of the calculated 0D trapped state dispersion and the measurement.** The ΔV for the simulation input is calibrated using the blue shifted repulsive polaron state.

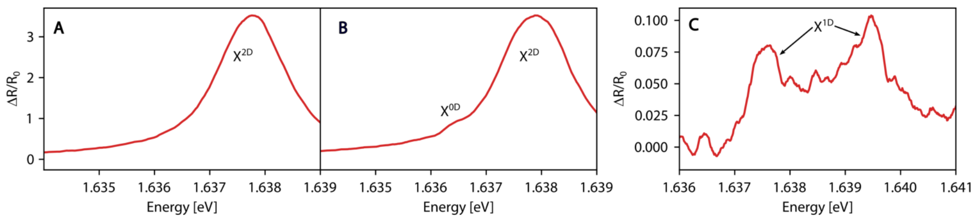

Figure S6: **RC spectra of 2D, 0D and 1D excitons.** (**A**) Spectrum at $V_R = -7.8\,\mathrm{V}$ from panel C in Fig. S9. (**B**) Spectrum at $V_R = -7.8\,\mathrm{V}$ from panel B in Fig. S9. (**C**) Spectrum at $V_R = -10.5\,\mathrm{V}$ from panel **B** in Fig. S10.

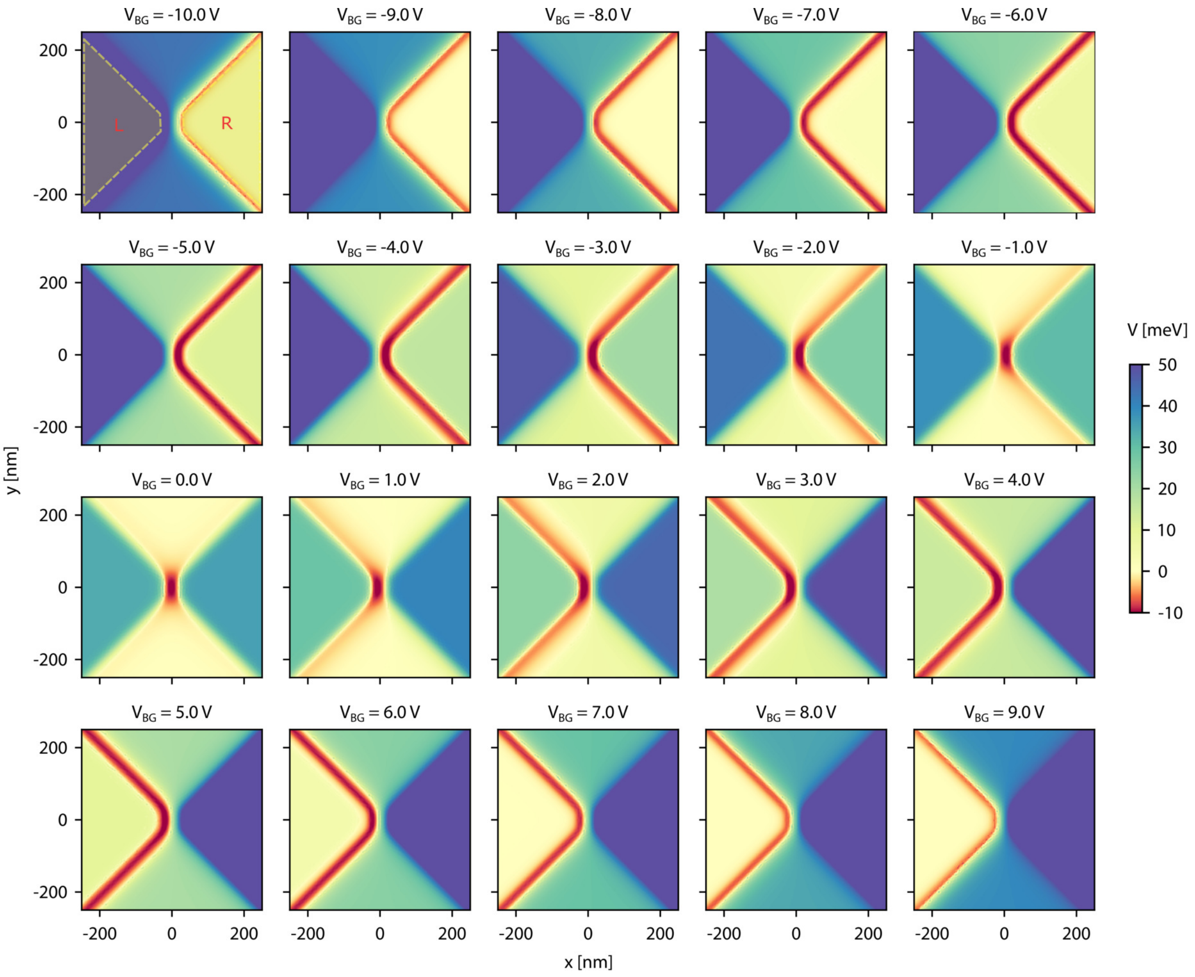

Figure S7: **Simulation results for the evolution of the potential landscape with the backgate voltage at** $(V_\mathrm{L}, V_\mathrm{R}) = (5\,\mathbf{V}, -5\,\mathbf{V})$**.** It shows a continuous transition from the 0D dot confinement ($V_\mathrm{BG} = 0\,\mathrm{V}$) to 1D edge confinement wrapped around the right (left) electrode when the backgate is tuned to negative (positive) voltage.

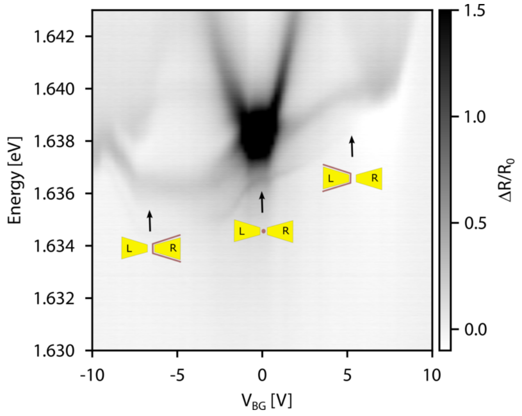

Figure S8: **Reflectivity spectra in the bow tie structure as a function of V_{BG} at $(V_{\mathrm{L}}, V_{\mathrm{R}}) = (5\,\mathbf{V}, -5\,\mathbf{V})$.** The asymmetry between positive and negative BG regions originates from the asymmetric doping behaviour of our device with respect to electron and hole injection.

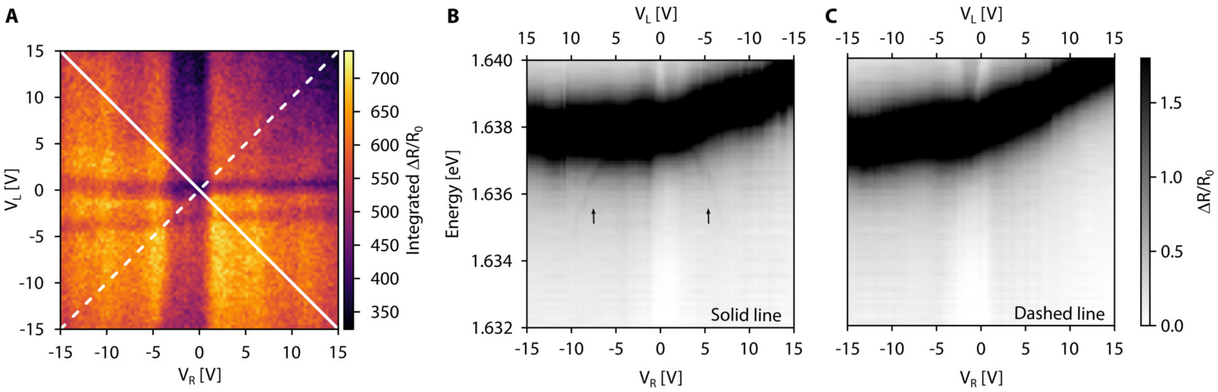

Figure S9: $V_{\mathrm{L}} - V_{\mathrm{R}}$ **dual gate scan map of reflectivity at $V_{\mathrm{BG}} = 0\,\mathbf{V}$.** (**A**) The integrated reflection map, the vertical (horizontal) stripe shows the gate range where the 2D exciton under the right (left) bow tie electrode is neutralized. (**B**) The $\Delta R/R$ line cut along the solid line in (**A**) which corresponds to applying ΔV across the bow tie. As expected, signature for 0D trapped state is present. (**C**) The $\Delta R/R$ line cut along the dashed line in (**A**) corresponding to $\Delta V = 0\,\mathrm{V}$, where no trapped state is observed.

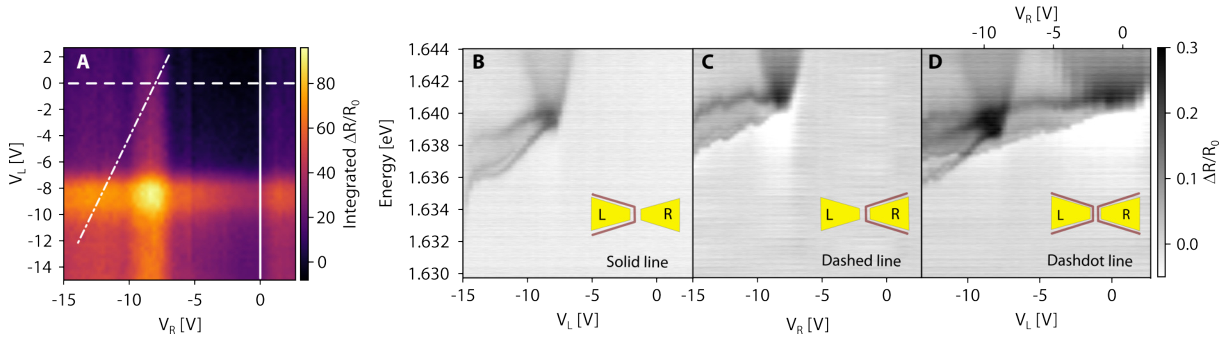

Figure S10: $V_{\mathrm{L}} - V_{\mathrm{R}}$ **dual gate scan map of reflectivity at** $V_{\mathrm{BG}} = 10$ **V.** (**A**) The integrated reflection map, the vertical (horizontal) stripes shows the gates range where the 2D exciton under the right (left) bow tie electrode is neutralized. (**B**) The $\Delta R/R$ line cut along the solid line at $V_{\mathrm{R}} = 0$ V, displaying 1D confined states around the left bow tie electrode (as shown in the inset). (**C**) The $\Delta R/R$ line cut along the dashed line at $V_{\mathrm{L}} = 0$ V, displaying 1D confined states around the right bow tie electrode. (**D**) The $\Delta R/R$ line cut along the dashdot line, showing 1D confined states around both bow tie electrode.

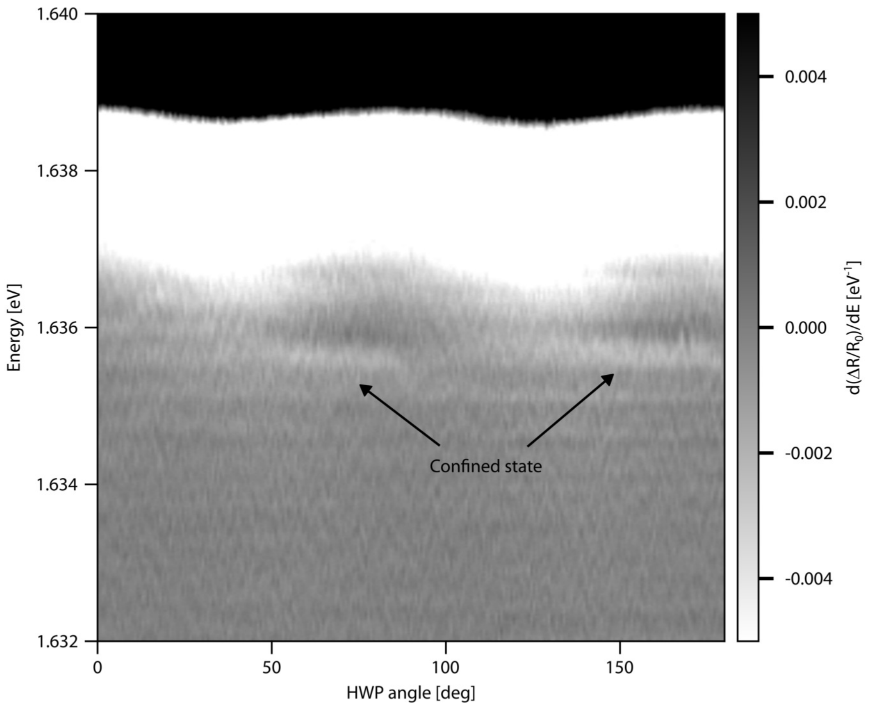

Figure S11: **Polarization dependence of the 0D trapped states in the bow tie structure.**

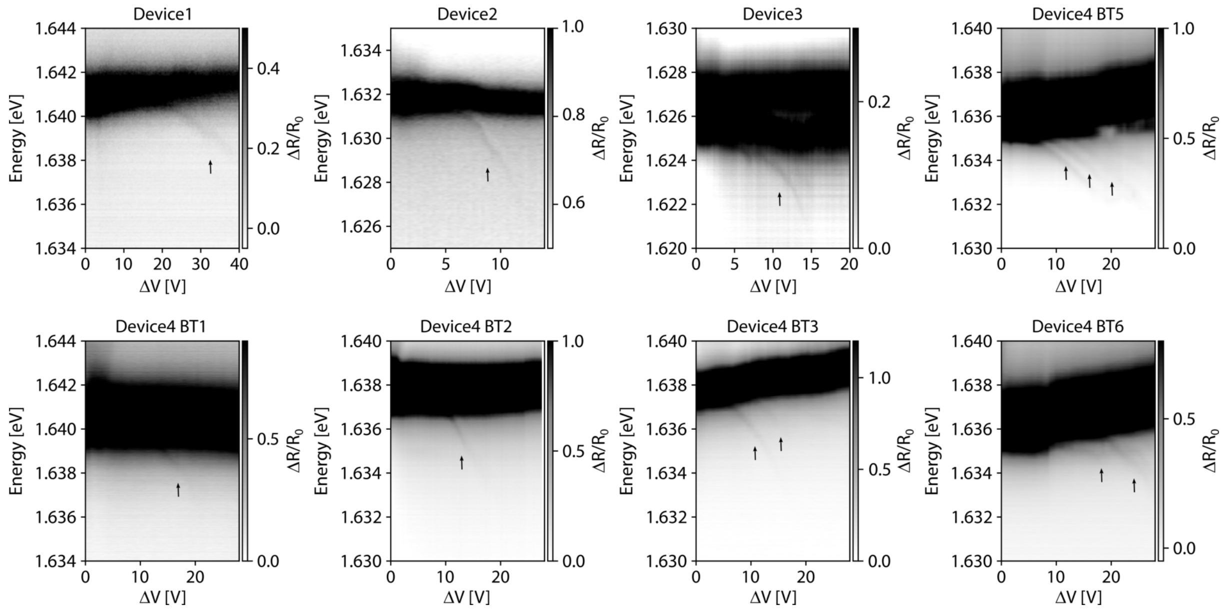

Figure S12: **0D confined states measured in bow tie structures on multiple samples.** The arrows point to the dispersing confined states.

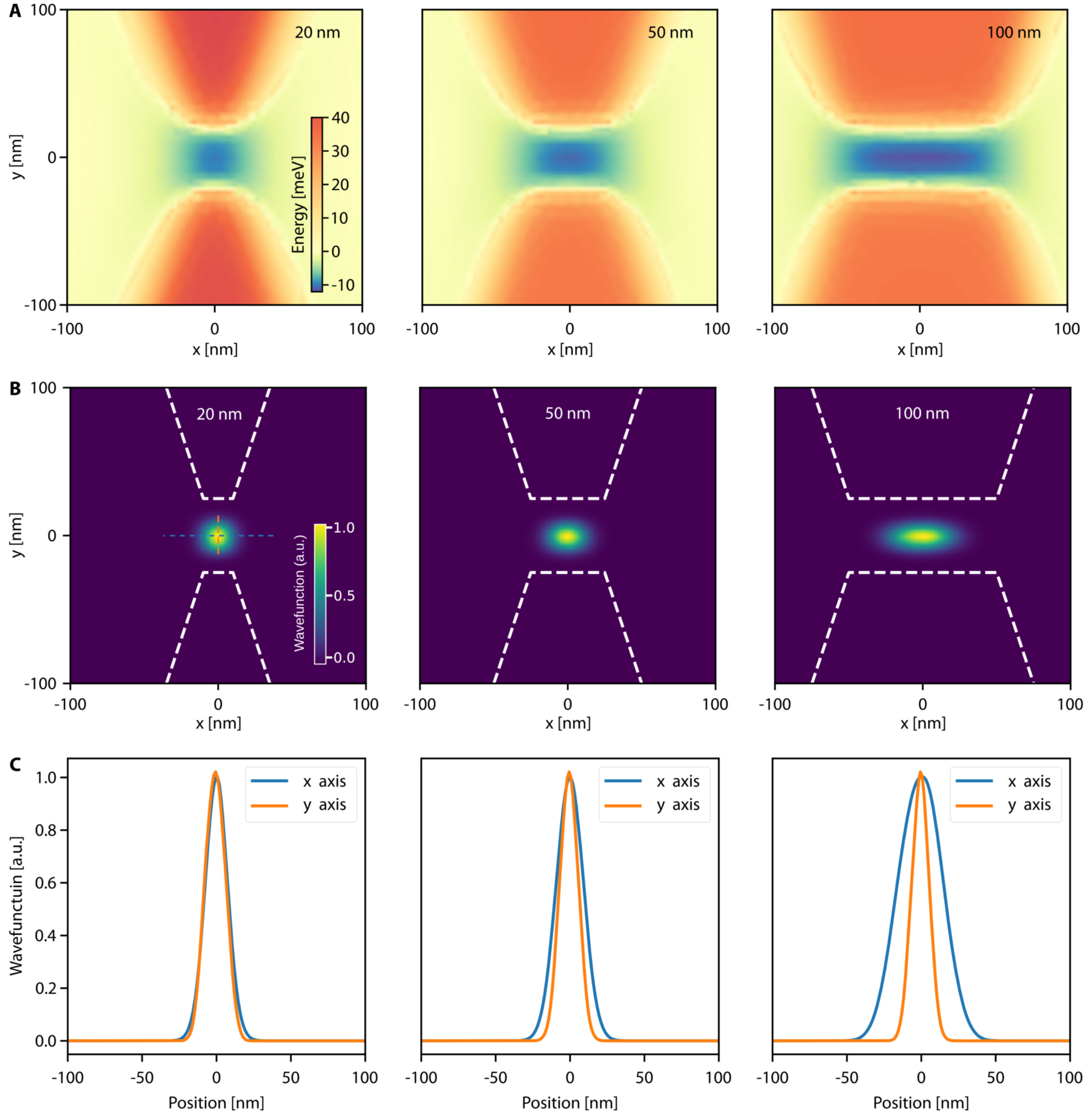

Figure S13: **Electrostatic simulation results on bow ties with different geometry.** The tip width of the bow tie is chosen to be 20 nm (left column), 50 nm (middle column) and 100 nm (right column), while the gap between the two bow tie is fixed to be 50 nm. For each geometry, we simulate (**A**) the total potential , (**B**) the ground state wavefunction and (**C**) the wavefunction cuts along x and y axis, which correspond to the blue and orange dashed line respectively in the first panel of (**B**). This shows that the confined state wavefunction can be set to symmetric or elongated forms depending on the bow tie geometry. The simulation is done with bow tie voltages +5 and -5 V.

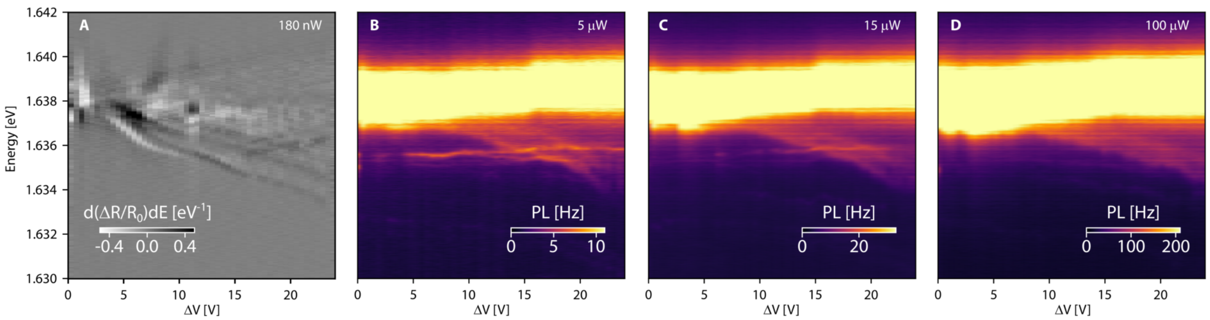

Figure S14: **Reflectivity and PL gate scans of 0D confined states in a 100 nm by 100 nm bow tie trap.** (**A**) First-derivative reflectance contrast spectra as a function of bow tie bias using 180 nW SLED power. Gate-dependent PL map, with excitation wavelength of 720 nm, and optical power of 5 uW (**B**), 15 uW (**C**) and 100 uW (**D**).

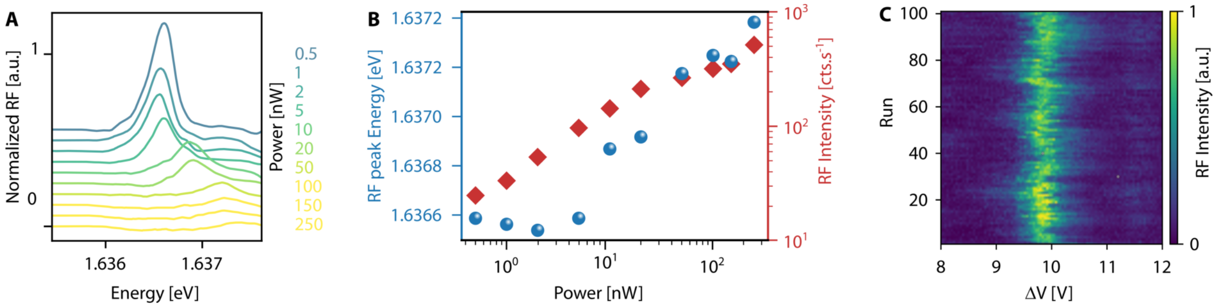

Figure S15: **Resonance fluorescence from the 0D confined states.** (**A**) Power dependent RF spectra of the 0D state under ps pulsed excitation. (**B**) Extracted central energy and integrated intensity of the RF signals as a function of pump power. (**C**) Stability of the RF signal acquired over 10 hours by scanning the gate voltage ΔV for a fixed CW illumination at 5 nW.
